# Investigating the contextual modulation of postoperative gastrointestinal motility: a methodological feasibility study combining a standardized intervention protocol with wireless motility capsule monitoring

**DOI:** 10.3389/fpsyg.2026.1913608

**Published:** 2026-10-08

**Authors:** Martin Coenen, Manfred Schedlowski, Gunther Hartmann, Jörg C. Kalff, Thomas Büttner, Tim O. Vilz

**Affiliations:** 1 Clinical Study Core Unit, Study Center Bonn (SZB), University Hospital Bonn, University of Bonn, Bonn, Germany; 2 Institute for Clinical Chemistry and Clinical Pharmacology, University Hospital Bonn, University of Bonn, Bonn, Germany; 3 Institute of Medical Psychology and Behavioral Immunobiology, Center for Translational Neuro- and Behavioural Sciences, University Hospital Essen, Essen, Germany; 4 Department of Clinical Neuroscience, Osher Center for Integrative Medicine, Karolinska Institutet, Stockholm, Sweden; 5 Department of Surgery, University Hospital Bonn, University of Bonn, Bonn, Germany; 6 Department of Urology and Paediatric Urology, University Hospital Bonn, University of Bonn, Bonn, Germany; 7 Department of General and Visceral Surgery, Charité—Universitätsmedizin Berlin, Corporate Member of Freie Universität Berlin und Humboldt-Universität zu Berlin, Campus Benjamin Franklin, Berlin, Germany

**Keywords:** feasibility, gastrointestinal motility, gut-brain axis, methodology, objective endpoints, placebo effect, SmartPill, therapeutic context

## Abstract

**Background:**

Objective measurement of placebo effects on gastrointestinal function remains a methodological challenge, as most studies rely on patient-reported outcomes. Wireless motility capsule technology (SmartPill®) offers continuous, objective, in-vivo recording of motility parameters throughout the gastrointestinal tract, potentially providing physiological endpoints for placebo research. This methodological feasibility study, nested within the prospective PIDuSA trial, aimed to evaluate whether SmartPill®-based motility monitoring can be feasibly combined with a standardized contextually-enhanced drug administration protocol in patients following abdominal surgery.

**Methods:**

A subgroup of six patients received laxatives (9 administrations) from a senior physician incorporating contextual moderators known to enhance placebo responses. Forty-three control group patients received standard administration by nursing staff (180 administrations). The SmartPill® continuously recorded gastrointestinal motility parameters. Primary outcome was the Motility Index calculated over three 30-min periods: baseline, early effect (30–60 min post-administration), and late effect (240–300 min post-administration). Clinical outcomes included stool passage and peristalsis development within 24 h.

**Results:**

The methodological approach proved feasible, although no consistent improvements in motility parameters were observed. The contextual intervention protocol was associated with descriptive trends toward improved clinical indicators like peristalsis on auscultation (77.8% vs. 55.6%) and stool passage (33.3% vs. 31.7%), but physiological parameters remained largely unchanged.

**Conclusion:**

This feasibility study demonstrates that combining a standardized contextual intervention protocol with continuous SmartPill®-based gastrointestinal motility monitoring is operationally feasible in the postoperative setting. The approach successfully captured objective physiological data across all administrations, providing a preliminary methodological framework for future adequately powered trials investigating the modulation of gastrointestinal function by therapeutic context interventions. The absence of significant motility changes in this exploratory sample size does not allow conclusions regarding the presence or absence of contextual effects. We provide a detailed protocol description to facilitate replication and inform future study design.

## 1 Introduction

The placebo effect is a well-documented phenomenon associated with beneficial outcomes independent of active pharmacological substances (Kaptchuk and Miller, 2015; Colloca and Barsky, 2020). Psychological processes including learning, conditioning, and expectations play a crucial role. They are all further shaped by contextual factors like the patient-provider relationship and the setting in which treatment is delivered, which constitute a potent non-pharmacological determinant of treatment outcomes (Finniss et al., 2010; Howe et al., 2017; Kaptchuk et al., 2008; Burke et al., 2026). The gut-brain axis is a bidirectional communication network integrating neural, hormonal, and immune signals (Mayer et al., 2022) through which psychological states can significantly influence gastrointestinal function, suggesting that targeted psychological interventions may modulate gastrointestinal activity (Flik et al., 2017; Kaptchuk et al., 2008; Mayer, 2011). It is therefore plausible that intestinal function may likewise be amenable to modulation through contextual effects. In gastroenterology, placebo interventions have been shown to modulate both gastrointestinal motility (Kaptchuk et al., 2010; Lembo et al., 2021; Meissner, 2009; Elsenbruch and Enck, 2015) and symptoms, particularly in irritable bowel syndrome and inflammatory bowel disease (Patel et al., 2005; Ilnyckyj et al., 1997; Su et al., 2004; Ford and Moayyedi, 2010). Despite its relevance, the role of contextual and placebo effects in pharmacological treatment of gastrointestinal disorders remains underexplored, particularly for drugs supporting bowel movements whose efficacy generally remains limited (Traut et al., 2008).

A critical methodological gap in placebo research is the frequent reliance on patient-reported outcomes, which are inherently susceptible to expectation bias and thus confound the very effects being studied. The true impact on objectively measured physiological processes is therefore usually not directly analyzed in this context. Wireless motility capsule technology (SmartPill®) allows continuous, objective assessment of gastrointestinal motility through real-time recording of intraluminal pressure, pH, and temperature throughout the gastrointestinal tract (Rao et al., 2009). Here, we present the feasibility of combining two novel methodological approaches, each of which may constitute an important instrument for the investigation of psychological and pharmacological influences on the gut–brain axis: (1) the use of the SmartPill® to obtain objective gastrointestinal parameters for investigating contextual effects, and (2) a detailed standardized contextual intervention protocol as a basis for future clinical trials to investigate placebo effects in the gastrointestinal tract. To this end, a subgroup of patients of the PIDuSA trial (Vilz et al., 2016) received laxatives via a structured, contextually-enhanced administration protocol delivered by a designated senior physician rather than nursing staff. The protocol operationalized multiple established contextual moderators of the placebo response—including physician status, attire, empathic engagement, provider warmth, and positive verbal outcome framing (Di Blasi et al., 2001; Blasini et al., 2018; Howe et al., 2017; Rehman et al., 2005)—into a reproducible behavioral script constituting an “enhanced therapeutic context.” As we recently showed that administration of prokinetics and laxatives did not induce significant improvements in bowel motility (Coenen et al., currently under review) we additionally aimed to generate preliminary descriptive data on potential motility changes in gastrointestinal activity induced by the contextual intervention protocol measured by the SmartPill®.

## 2 Methods

### 2.1 The PIDuSA trial

PIDuSA was a prospective, open-label medical device trial. Primary endpoints were safety and suitability of the wireless motility capsule device SmartPill® (Medtronic, Minneapolis, Minnesota, USA) for use in the context of abdominal surgery. For details concerning study procedures, trial management, data handling and ethical standards please refer to the detailed study protocol published previously (Vilz et al., 2016). In brief, 49 patients undergoing abdominal surgery at high risk of postoperative ileus (POI) were included in the study between January 2015 and November 2017. Main exclusion criteria comprised emergency interventions, condition after radiation therapy at the site of operation in the last 2 months, known dysphagia, non-steroidal anti-inflammatory drug (NSAID) enteropathy in the past, body mass index (BMI) > 40, patients with active implantable devices, medication with proton pump inhibitors (PPIs), antacids or H2 blockers, reflux oesophagitis grade III and IV according to Savary and Miller, esophagojejunostomy, fistulas of the esophagus and/or stomach, which cannot be repaired during surgery, known or suspected stenoses fistulas of the gastrointestinal tract, not being repaired by the intervention, active Crohn’s disease (Crohn’s Disease Activity Index (CDAI) > 450), pronounced diverticulosis or diverticulitis not being resected during the operation, or patients with several abdominal surgical interventions with increased risk of anastomotic leakage (e. g. required immunosuppression), all of which associated with an increased risk for capsule retention. During surgery, a SmartPill® was placed into the stomach that captured data on gastrointestinal (GI) motility data until excreted. In a non-surgical setting, the patients would simply swallow the SmartPill on their own under supervision with a few sips of water. Daily study visits conducted each morning included a physical examination and assessed whether patients had a bloated abdomen and had passed stools, their current oral food tolerance and the presence of peristalsis on auscultation. The SmartPill® demonstrated adequate safety in this setting, the primary objective of PIDuSA, as already published (Van Beekum et al., 2021). The study was approved by the independent ethics committee of the University of Bonn on April 16th 2014 (092/14-MPG).

### 2.2 Contextual intervention protocol

In the PIDuSA trial, laxatives and prokinetic agents were administered by nursing staff as part of standard postoperative care. To investigate whether contextual factors with the activation of placebo mechanisms via the gut–brain axis can augment the effects of laxatives and prokinetic agents on gastrointestinal motility, a subgroup of patients was allocated to the therapeutic context protocol determined solely by the availability of the study physician at the time of drug administration and received their medication under highly standardized and contextually-enhanced conditions (*contextual intervention group*). No formal randomization procedure was employed for this substudy.

The following protocol describes the steps of a structured behavioral script which must be executed sequentially for each eligible patient encounter. The interaction was deliberately structured to incorporate a competent demeanor, appreciative interaction and communication, and positive verbal framing as contextual elements known to modulate treatment responses (Wager and Atlas, 2015; Howe et al., 2017; Blasini et al., 2018):

1 Physician preparation (pre-encounter): The designated physician, holding at least the rank of a senior physician wears a white medical coat and a name badge explicitly indicating the actual professorial academic title (e.g., “Prof. Dr.”), ensuring full professional attire and exuding competence prior to entering the patient room.
2 Patient greeting and rapport establishment: The physician enters the room calmly, greets the patient by name, provides a formal self-introduction including title and role of an experienced senior physician, and initiates a brief, empathic inquiry into the patient’s current wellbeing conveying a high level of authority, competence, and calm professionalism. Sustained eye contact is maintained throughout. Verbal delivery is measured, warm, and unhurried.

Open questioning: The physician explicitly invites the patient to voice concerns or questions, demonstrating attentive listening. Sufficient time is allocated for the patient to respond; interruptions are to be avoided.

3 Explanation of pharmacological effects and positive outcome framing: Immediately prior to drug administration, the physician delivers a structured verbal explanation of the drug’s mechanism of action, tailored to the patient’s health literacy. The explanation explicitly emphasizes the agent’s established pharmacological efficacy and the physician’s positive clinical experience with prior patients. Language is accessible, confident, and affirmative. The verbal interaction is intentionally framed to emphasize anticipated therapeutic benefit without drawing attention to possible side effects.
4 Drug administration: The designated laxative or prokinetic agent is administered by the physician personally—handing out oral medication or connecting and starting parenteral infusion, maintaining direct patient contact throughout.
5 Closing the encounter: The physician concludes the interaction with a positive and reassuring farewell, reiterating confidence in the anticipated therapeutic effect. The tone remains calm, professional, and encouraging throughout.

#### 2.2.1 Timing

Total estimated duration of the standardized encounter: approximately 10 min per patient. Individual components are timed as follows:

- Step 1—Physician preparation: ~2 min (outside patient room)
- Steps 2—Greeting, rapport, open questioning: ~3 min
- Steps 3—Pharmacological explanation and benefit framing: ~3 min
- Step 4—Drug administration: ~1 min
- Step 5—Closing: ~1 min

### 2.3 Patient subgroups

The procedures and analyses have been described previously (Vilz et al., 2016). Participants of the PIDuSA trial meeting the POI criteria of Vather et al. (2013) were treated with intravenous prokinetics (250 mg Erythromycin, 10 mg MCP or 10 mg MCP + 0.5 mg Neostigmine) or enteral laxatives (5 mg oral/10 mg rectal Bisacodyl or 15 g of MgSO4) during the trial based on clinical considerations. Baseline variables (Age, Body-Mass-Index [BMI] and gender) were extracted from the electronic case report form (eCRF). For this substudy a subgroup of patients of the PIDuSA trial were randomly selected depending on the availability of the study physician and received their medication under highly standardized and contextually enhanced conditions (*contextual intervention group*).

### 2.4 Outcome measures and validation

For the analysis we processed the GI data continuously recorded by the SmartPill® using the manufacturer-provided software (MotiliGI, v3.0, Medtronic, Minneapolis, USA). A contraction leads to a temporary increase in the pressure of the GI tract by an amplitude between 10 and 300 mmHg. The Motility Index (MI) combines these pressure increases by the formula MI = loge(sum of amplitude × number of contractions + 1)/time and was employed as the primary outcome measure of this analysis (Camilleri et al., 1985). Additional outputs of the capsule included maximum pressure (in mmHg), mean number of contractions per minute (MC/min) and mean peak amplitude (MPA), defined as the sum of amplitudes divided by the number of contractions (in mmHg). To clinically validate these variables, we extracted data over a 10-min period during the study visits at the time of physical examination and compared them with the clinically reported presence or absence of peristalsis on auscultation.

An event recorder logged each administration of prokinetics or laxatives. As primary study readout MI was averaged over three 30-min periods for each administration. A baseline measurement of the Motility Index (MI_0_), the mean number of contractions per minute (MC/min_0_), the Mean Peak Amplitude (MPA_0_), the highest (hp_0_) and lowest pressure (lp_0_) during the 30 min preceding administration, a first effect measurement (MI_30_, MC/min_30_, MPA_30_, hp_30_, lp_30_) covering 30 to 60 min after administration and a second effect measurement (MI_240_, MC/min_240_, MPA_240_, hp_240_, lp_240_) during the 30 min 240 to 300 min after administration. Subsequently, Delta MI was calculated as difference between these periods as MI_30-0_ = MI_30_ − MI_0_ and MI_240-0_ = MI_240_ − MI_0_. Further information from the accompanying daily study visits including days after surgery, opioid usage, oral dietary intake, physical examination findings, stool passage were taken from the eCRF and assigned to each administration.

For the purposes of this feasibility substudy, the following operational indicators were used to assess feasibility: (1) successful completion of the full therapeutic context protocol as specified, (2) completeness of SmartPill® recordings, (3) absence of protocol deviations, and (4) absence of capsule-related adverse events. Given the exploratory nature of this nested substudy, these specific feasibility indicators were defined retrospectively and were not formally pre-specified in the main trial protocol.

### 2.5 Statistics

The sample size of 55 patients undergoing abdominal surgery having a high risk for POI was calculated for the primary endpoint of the PIDuSA trial and suggested that events with a probability of 3% could be seen with a certainty of 80% for at least once in the sample. For this substudy descriptive statistics included frequencies and proportions for categorical variables while medians and interquartile ranges (IQR) were reported for continuously coded variables. Due to the small sample size of the *contextual intervention group* (*n* = 6 patients, 9 administrations) and the heterogeneity of the administered medications regarding their mechanisms of action, target organs, and routes of administration, outcomes for this group were evaluated and reported purely descriptively. Consequently, inferential statistical testing (e. g. calculation of *p*-values) was omitted for comparisons involving the *contextual intervention group*. All data were coded and analyzed using RStudio 2024.04.02 + 764^1^ using the packages “tidyverse” (v.2.0.0), “Table 1” (v1.4.3) and “lmerTest” (v3.1–3) and visualized by the R packages ggplot2 (v3.5.1) and ggpubr (v.0.6.0).

**Table 1:** Outcomes of the drug administrations.

	Control (*N* = 180)	Contextual intervention (*N* = 9)
Drug cohort		
Laxatives	75 (41.7%)	7 (77.8%)
Prokinetics	105 (58.3%)	2 (22.2%)
Baseline motility index		
Mean (SD)	3.91 (4.10)	6.84 (4.30)
Median [Min, Max]	3.02 [0, 13.3]	6.34 [0, 12.5]
Clinical findings		
Peristalsis	60 (33.3%)	2 (22.2%)
Bloated abdomen	68 (37.8%)	0 (0%)
Abdominal pain	5 (2.8%)	1 (11.1%)
Stools passed yet	51 (28.3%)	1 (11.1%)
Drugs		
Bisacodyl oral (5 mg)	7 (3.9%)	0 (0%)
Bisacodyl rectal (5 mg)	29 (16.1%)	1 (11.1%)
Erythromycin i.v. (250 mg)	57 (31.7%)	0 (0%)
MCP i.v. (10 mg)	14 (7.8%)	2 (22.2%)
MCP + Neostigmine i.v. (10 mg + 0.5 mg)	34 (18.9%)	0 (0%)
MgSO4 oral (15 g)	39 (21.7%)	6 (66.7%)

Clinical findings and measurements and drugs separated for the control and the contextual intervention group.

## 3 Results

### 3.1 Patient characteristics

The full contextual intervention protocol was successfully applied to six patients during 9 administrations as specified: one patient received 5 mg rectal Bisacodyl, five patients received six administrations of 15 g oral MgSO4, one of whom also received 10 mg intravenous MCP twice. SmartPill® recordings were obtained without interruption across all observation windows. No protocol deviations or capsule-related adverse events occurred in this subgroup. *Control group* patients received standard administration by nursing staff (43 patients, 180 administrations). At the timepoint of drug administration, no patient in the *contextual intervention group* exhibited a bloated abdomen on clinical examination, one patient suffered from abdominal pain, seven of nine showed an absence of peristalsis on auscultation, and eight patients had not yet passed stools. These results are summarized in Table 1.

### 3.2 Measurements of motility capsule

SmartPill® recordings were successfully obtained before and after all included administrations. As recently shown (Coenen et al., currently under review), the standard administration of prokinetics and laxatives did not induce any significant or consistent directed changes to the main outcome measure of the *motility index* (MI), indicating that the administration did not induce improvements in bowel motility. The application of the contextual intervention protocol also showed no directed changes of the measured parameters compared to the standard administration in the *control group*. Paired plots comparing measurements before and after administration are shown in Figure 1A even indicating reductions of gastrointestinal motility in seven of 9 administrations. Descriptive analysis of the subgroup receiving MgSO4 also did not reveal consistent trends of the contextual intervention protocol on motility parameters (data not shown).

**Figure 1:**
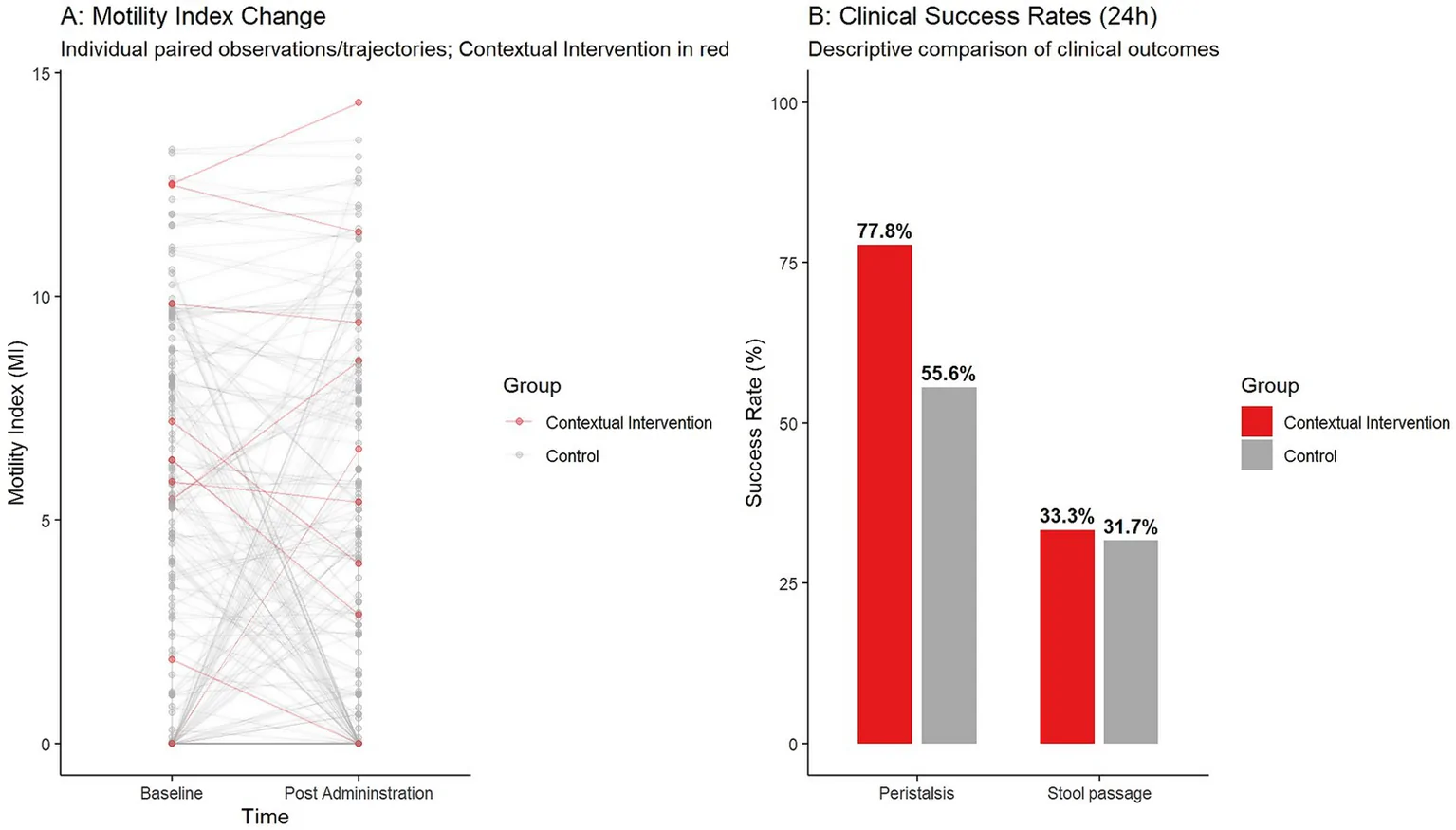
Effects of prokinetics and laxatives in the control and the contextual intervention group **(A)** Paired line plot of Motility Index (MI) comparing MI before and after administration and **(B)** outcomes on the main clinical endpoints stool passage and peristalsis within 24 h.

### 3.3 Clinical findings

A descriptive comparison of clinical outcomes within the first 24 h following drug administration revealed that 33.3% of administrations (3 of 9) in the *contextual intervention group* were followed by stool passage within 24 h, compared to 31.7% (57/180 administrations) in the *control group* (Figure 1B). Looking at findings of the physical examination, 77.8% (7/9) of patients in the *contextual intervention group* developed peristalsis compared to 55.6% (100/180) of the *control group* (Figure 1B). The small sample size precludes inferential statistical testing. Therefore these data provide descriptive trends only and show the practical feasibility of combining contextual clinical interventions with objective motility monitoring for future hypothesis-driven studies.

## 4 Discussion

In this study we present the combination of two novel methodological approaches, each of which may constitute an important building block for the investigation of psychological and pharmacological influences on the gut-brain axis. To our knowledge, this is the first study to combine a standardized, operationally defined contextual intervention protocol with the continuous, objective measurement of GI motility in postoperative patients. The central finding of this feasibility study is that such a combination is practicable, and capable of generating continuous physiological data—demonstrating that the methodological approach itself is viable as a basis for future adequately powered trials. Placebo responses in gastrointestinal disorders range from 20 to 40% (Ilnyckyj et al., 1997; Su et al., 2004; Ford and Moayyedi, 2010). While most studies focus on subjective endpoints (Musial et al., 2007; Bosman et al., 2021; Shah, 2021), the present study establishes the technical and operational feasibility of a standardized contextual intervention protocol combined with objective SmartPill® monitoring—offering a genuinely novel in-vivo framework to evaluate placebo effects with physiological precision.

The standardized protocol operationalized a multicomponent enhanced therapeutic context, incorporating physician interaction conveying competence and positive framing as known drivers of placebo responses (Kaptchuk and Miller, 2015; Petrie and Rief, 2019). In this pilot study the protocol did not result in consistent improvements in gastrointestinal motility, aligning with earlier findings of limited efficacy of prokinetics and laxatives on gastrointestinal motility *without* contextual interventions (Cheape et al., 1991; Orlando et al., 1994; Traut et al., 2008). Descriptive trends—higher proportion developing peristalsis within 24 h—should be interpreted with caution given the small and heterogenous sample, though it would be consistent with gut-brain axis influence (Mayer et al., 2022) and warrant further consideration. Although our results indicate contextual interventions may not be sufficient to produce measurable short-term effects on postoperative bowel motility early after surgery, the absence of significant findings in this exploratory sample size does not allow conclusions regarding the presence or absence of contextual effects.

A notable strength of the study is the use of the SmartPill® to provide objective physiological endpoints. Most placebo research in gastrointestinal disorders relies on patient-reported outcomes susceptible to expectation bias (Irvine et al., 2016; Bosman et al., 2021). Wireless capsule technology allowed direct quantification of peristaltic activity, making this one of few studies evaluating placebo effects with physiological measures. This methodological innovation offers a promising path for future trials aiming to disentangle the physiological effects of therapeutic context interventions on the gastrointestinal tract.

Limitations include the very limited sample size, open-label design, and pragmatic allocation introducing potential selection bias. The small sample size for different pharmacological agents and types of surgeries limited statistical power to detect subtle yet potentially clinically relevant changes in objective parameters, particularly, as the contextual intervention occurred in an environment with high variability in surgical trauma, inflammation, recovery, and co-treatment, potentially attenuating effects. Importantly, there is substantial heterogeneity in the pharmacological treatments administered. During the trial prokinetics and laxatives with distinct routes of administration, mechanisms of action and target segments with consequently different times of onset were administered at the investigator’s discretion. Specifically, prokinetics primarily stimulate the upper gastrointestinal tract (Malagelada and Malagelada, 2017), Bisacodyl is a stimulant laxative targeting the large bowel (Corsetti et al., 2021), and MgSO4 acts as an osmotic agent in the small and large bowel also indirectly stimulating gut motility (Vu et al., 2000). Furthermore, patients undergoing different types of abdominal surgery with variable courses of postoperative bowel recovery were included. Grouping these types of pharmacological treatments and abdominal surgeries together represents an important confounder when comparing motility outcomes. The two groups additionally differed substantially in medication composition, with laxatives accounting for 77.8% of administrations in the *contextual intervention group* compared to 41.7% in the *control group*, and baseline Motility Index was notably higher in the *contextual intervention group* (median 6.34 vs. 3.02). These imbalances are a direct consequence of the pragmatic allocation and limit the interpretability of the between-group comparisons. However, the primary objective of this methodological feasibility study was to establish the operational viability of combining the standardized protocol with SmartPill® monitoring rather than to definitively prove clinical efficacy.

The intervention simultaneously modified multiple components of the therapeutic encounter—physician status and attire, empathic engagement, interpersonal attention, and positive verbal framing—constituting an “enhanced therapeutic context (Di Blasi et al., 2001; Blasini et al., 2018; Kaptchuk et al., 2008; Rehman et al., 2005).” Future studies should incorporate expectancy instruments to test expectation as a mechanistic mediator of any motility changes observed. Finally, specific motility parameters might not fully capture functional recovery as GI motility is complex and multifactorial (Browning and Travagli, 2014), and readouts focused on short-term changes, whereas contextual effects may require longer durations. Of note, the SmartPill® does not directly measure peristalsis and cannot distinguish between contractile activity and effective coordinated peristaltic contraction that results in propulsion of intestinal contents (Grønlund et al., 2017), which should be considered when interpreting the clinical significance of observed motility changes. Nevertheless, wireless motility capsule monitoring ranks among the most established, minimally invasive technologies available for continuous, physiological in-vivo assessment of intraluminal contractile dynamics throughout the entire gastrointestinal tract.

In summary, this feasibility study demonstrates that the combination of a standardized contextual intervention protocol with SmartPill®-based objective GI motility monitoring is operationally viable, and capable of generating objective physiological data. The operationalized protocol described here—including physician attire, interaction structure, language, and timing—provides a reproducible template for future trials employing a pre-registered, randomized design with prospectively defined feasibility and efficacy endpoints. The gut-brain axis remains compelling, and placebo mechanisms—while difficult to capture—may exert influence in larger and more homogeneous cohorts, over longer periods, or with supportive measures.

## Data Availability

The raw data supporting the conclusions of this article will be made available by the authors, without undue reservation.

## Footnotes

1 https://CRAN.R-project.org/.
